# Anogenital Distance and Penile Length in Infants with Hypospadias or Cryptorchidism: Comparison with Normative Data

**DOI:** 10.1289/ehp.1307178

**Published:** 2013-12-06

**Authors:** Ajay Thankamony, Ngee Lek, Dan Carroll, Martyn Williams, David B. Dunger, Carlo L. Acerini, Ken K. Ong, Ieuan A. Hughes

**Affiliations:** 1 Department of Paediatrics, University of Cambridge, Addenbrooke’s Hospital, Cambridge, United Kingdom; 2 Department of Paediatric Surgery and Urology, Addenbrooke’s Hospital, Cambridge University Hospital NHS Trust, Cambridge, United Kingdom; 3 MRC (Medical Research Council) Epidemiology Unit, Institute of Metabolic Science, University of Cambridge, Addenbrooke’s Hospital, Cambridge, United Kingdom

## Abstract

Background: Anogenital distance (AGD) in animals is a sensitive biomarker of fetal endocrine disruption and the associated testicular dysgenesis syndrome (TDS). However, AGD in human infants with cryptorchidism and hypospadias, which are potential manifestations of TDS during childhood, is not clearly described.

Objective: Our aim was to compare AGD in boys with cryptorchidism or hypospadias against normative data.

Methods: Boys with isolated cryptorchidism (*n* = 71, age 13.4 ± 5.8 months) or hypospadias (*n* = 81, age 11.4 ± 6.2 months) were recruited from a tertiary center for measurement of AGD and penile length; they were compared with 487 healthy full-term boys from a birth cohort by deriving age-specific standard deviation scores (SDS).

Results: Boys with cryptorchidism were older (*p* = 0.048) compared with boys with hypospadias. Boys with hypospadias had shorter mean AGD and penile length SDS than healthy boys (both *p* < 0.0001). Mean AGD and penile length SDS values in boys with cryptorchidism were longer than mean values in boys with hypospadias (both *p* < 0.01) and shorter than mean values in healthy boys (both *p* < 0.0001). Mean penile length SDS decreased as the severity of hypospadias increased (*p*_trend_ = 0.078).

Conclusions: In the study population, AGD and penile length were reduced in boys with hypospadias or cryptorchidism relative to normative data derived from a longitudinal birth cohort. The findings support the use of AGD as a quantitative biomarker to examine the prenatal effects of exposure to endocrine disruptors on the development of the male reproductive tract.

Citation: Thankamony A, Lek N, Carroll D, Williams M, Dunger DB, Acerini CL, Ong KK, Hughes IA. 2014. Anogenital distance and penile length in infants with hypospadias or cryptorchidism: comparison with normative data. Environ Health Perspect 122:207–211; http://dx.doi.org/10.1289/ehp.1307178

## Introduction

Declining secular trends in male reproductive health with increasing incidence of cryptorchidism, hypospadias, testicular cancer, and reduced sperm quality have been reported by several epidemiological studies in many countries (Acerini and Hughes 2006; Diamanti-Kandarakis et al. 2009; Toppari et al. 2010; Wohlfahrt-Veje et al. 2009b). Geographical variation in the incidences of these conditions suggests exposure to environmental agents as a possible causative factor (Boisen et al. 2004). Furthermore, the four disorders are associated with each other and postulated to be the manifestation of an underlying entity known as testicular dysgenesis syndrome (TDS). Hypospadias and cryptorchidism are potential manifestations of TDS at birth. Exposure to environmental chemicals that act as endocrine disruptors has been proposed as one of the pathogenetic mechanisms underlying abnormal fetal testicular development that characterizes TDS (Skakkebaek et al. 2001; Wohlfahrt-Veje et al. 2009b). This is supported by several animal studies (Dean et al. 2012; van den Driesche et al. 2012). Measurement of anogenital distance (AGD) has been proposed as a quantitative biomarker of fetal endocrine disruptor exposure in humans (Arbuckle et al. 2008).

AGD is a marker of perineal growth and caudal migration of the genital tubercle, and is androgen dependent in male rodents (Bowman et al. 2003). In animal studies, AGD measured from the genital tubercle to the anus is a sensitive marker of *in utero* exposure to androgens and anti-androgens, and is used extensively in animal reproductive toxicology studies (McIntyre et al. 2001). Shorter AGD in human infants has been associated with prenatal exposure to a variety of environmental chemicals (Miao et al. 2011; Swan et al. 2005; Torres-Sanchez et al. 2008). Reduced AGD has also been proposed as a marker of testicular dysfunction in adult men (Eisenberg et al. 2011; Mendiola et al. 2011). Establishing the alterations in AGD in cryptorchidism and hypospadias—the most common genital anomalies at birth in boys—is important in determining the role of AGD as a biomarker of fetal endocrine disruption and TDS in humans.

Some cross-sectional studies have reported shorter weight-adjusted AGD in boys with cryptorchidism, and shorter AGD in boys with hypospadias (Hsieh et al. 2008, 2012; Swan et al. 2005). However, the studies relied on derivatives of AGD to adjust for age-related changes in AGD in the absence of normative data (Swan 2006) and used measurements performed under anesthesia (Hsieh et al. 2008, 2012). Recently we and others have published normative data for AGD during infancy based on large population studies (Papadopoulou et al. 2013; Thankamony et al. 2009). Both studies reported a characteristic nonlinear pattern of rapid growth in the first year and little change thereafter. Therefore, applying normative data is useful in further characterizing AGD in these disorders, as highlighted in a recent review (Dean and Sharpe 2013). In the present study, we compared age-specific standard deviation scores (SDS) for AGD and penile length in boys with isolated cryptorchidism or cryptorchidism to normative data from a cohort of healthy boys.

## Methods

*Study population*. Boys < 2 years of age with isolated hypospadias or cryptorchidism were recruited from pediatric surgery outpatient and pre-operative assessment clinics at Cambridge University Hospital NHS Foundation Trust, Cambridge, United Kingdom, between 2010 and 2012. Children with anogenital malformations, which prevented identification of anatomical landmarks for measuring AGD, and in whom genital anomalies were a part of a malformation syndrome, were excluded. Controls were healthy full term-born boys (born > 37 weeks, birth weight > 2,500 g) from the Cambridge Baby Growth Study (CGBS) who had normal genitalia. In brief, CBGS is a longitudinal study established in 2001 to characterize hormonal, genetic, and environmental influences on infant growth and early male reproductive development (Thankamony et al. 2009). Measurement of AGD was included in the CBGS study protocol from 2006 onward. Mothers gave written informed consent for their infants to participate in the study. The research protocol was approved by the Cambridge Local Research Ethics Committee, and the study was conducted in accordance with the International Conference on Harmonization standards for Good Clinical Practice.

*Measurements.* Infants in the CBGS were measured at birth and at 3, 12, 18, and 24 months of age by trained pediatric nurses (Thankamony et al. 2009). Hypospadias and cryptorchidism cases had one set of AGD, weight, body length, and penile length measurements taken before age 2 years when they attended outpatient clinics. The same trained nurses performed the measurements in both cases and controls using the same protocols, which have been reported previously (Thankamony et al. 2009). Briefly, AGD was measured from the center of the anus to the junction between smooth perineal skin and rugated skin of the scrotum using Vernier calipers (DialMax; Wiha Premium Tools, Schonach, Germany). Penile length was measured from the lower edge of the pubic bone to the tip of the flaccid penis using Vernier calipers. Three consecutive measurements were taken at each assessment, and the average was used for analysis.

*Statistics.* Because the AGD and penile length increase substantially with age during early infancy, we generated normative data for AGD and penile length from the CBGS and calculated age-adjusted SDS. Reference centile curves were computed with the LMS (lambda-mu-sigma) method by Cole (Cole et al. 2011), using the software LMSchartmaker Light (Cole et al. 2011; Pan and Cole 2011). The LMS method is based on the use of Box–Cox transformations to normality through the calculation of a skewness parameter. The LMS parameters are the power in the Box–Cox transformation (L), the median (M), and the generalized coefficient of variation (S). Given these parameters and the assumption that the residuals follow a normal distribution, any desired percentile can be calculated. The LMS values for AGD and penile length derived from CBGS were used to calculate age-specific SDS employing the LMSgrowth software (Cole et al. 2011; Pan and Cole 2012). Age- and gender-specific SDS for weight and body length measurements were calculated by comparison to UK normative data (Freeman et al. 1995). All SDS calculations were adjusted for gestational age at birth. In CBGS boys who underwent longitudinal assessments, an average of the age and SDS of the measurements across multiple visits was used for analysis. Paired outcomes between the groups were compared using Student’s *t*-test. AGD is associated with weight (Swan et al. 2005); hence, weight SDS was used as a covariate in multivariable linear models (version 18.0; SPSS for Windows, IBM, Chicago, IL, USA). An association between penile length and severity of hypospadias based on the position of urethral meatus was reported in adults (Bracka 1989). Therefore, we evaluated linear trends in AGD and penile length among the subgroups of hypospadias with increasing grades of severity (glanular/subcoronal, penile, and perineal) by coding the subgroups using integer scores (1, 2, and 3 respectively) and analyzing using one-way analysis of variance (ANOVA). The data are expressed as mean ± SD unless otherwise specified. Also included (see Supplemental Material, Table S1) are LMS values of AGD and penile length derived from the CBGS at monthly intervals, which can be used to generate SDS in other populations using freely available LMSgrowth software (Cole et al. 2011; Pan and Cole 2012).

## Results

Data were collected in 154 boys with genital abnormalities; two boys had both hypospadias and cryptorchidism and were excluded from the study. The details of the remaining 81 boys with hypospadias, 71 with cryptorchidism, and 487 healthy boys from CBGS are shown in Table 1. Most of the healthy boys (96.1%), boys with hypospadias (94.4%), and boys with cryptorchidism (94.8%) were Caucasian. Boys in the CBGS were measured longitudinally on 3.5 ± 1.5 occasions.

**Table 1:** Characteristics of healthy controls and patients with cryptorchidism and hypospadias (mean ± SD or *p*-value).

Characteristic	Healthy boys^*a*^	Cryptorchidism	Hypospadias	Cryptorchidism vs. healthy boys	Hypospadias vs. healthy boys	Cryptorchidism vs. hypospadias
Observations (*n*)	487	71	81
Gestational age (weeks)	40.05 ± 1.20	38.95 ± 2.60	38.26 ± 3.27	< 0.0001	< 0.0001	0.21
Birth weight SDS	0.03 ± 0.88	–0.07 ±1.12	–0.39 ± 1.37	0.47	0.001	0.26
Age (months)	11.46 ± 6.23	13.43 ± 5.79	11.45 ± 6.15	0.012	0.98	0.048
Weight (kg)	8.81 ± 2.70	10.30 ± 2.02	9.15 ± 2.71	—	—	—
Body length (cm)	71.77 ± 10.21	76.16 ± 6.87	73.42 ± 10.16	—	—	—
AGD (mm)	29.75 ± 6.97	29.09 ± 6.78	24.65 ± 6.27	—	—	—
Penile length (mm)	36.09 ± 5.17	35.30 ± 5.89	28.70 ± 7.48	—	—	—
Weight SDS	0.01 ± 0.96	0.15 ± 1.26	–0.21 ± 1.46	0.24	0.087	0.037
Body Length SDS	0.29 ± 0.93	0.21 ± 1.49	0.10 ± 1.43	0.52	0.12	0.66
AGD SDS^*b*^	0.03 ± 0.77	–0.48 ± 0.93	–0.90 ± 0.89	< 0.0001	< 0.0001	0.005
Penile length SDS^*b*^	–0.02 ± 0.82	–0.35 ± 1.03	–1.34 ± 1.28	0.002	< 0.0001	< 0.0001
—, comparison not performed. ^***a***^The values of healthy boys were derived from the average of measurements/SDS values across multiple visits. Because the measurements vary with age, only the SDS values of the ­measurements were analyzed. ^***b***^Adjusted for weight SDS.						

**Figure 1:**
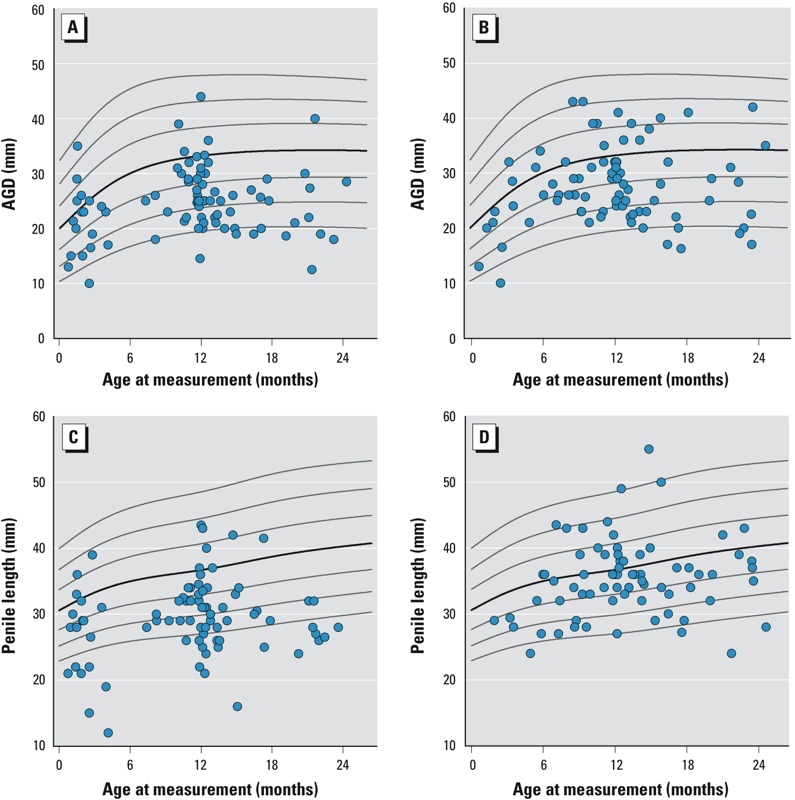
Distribution of AGD and penile length in boys with hypospadias (*A*,*C*) or cryptorchidism (*B*,*D*) against centile lines (3rd, 10th, 25th, 50th, 75th, 90th, and 97th centiles) from normative data.

**Figure 2:**
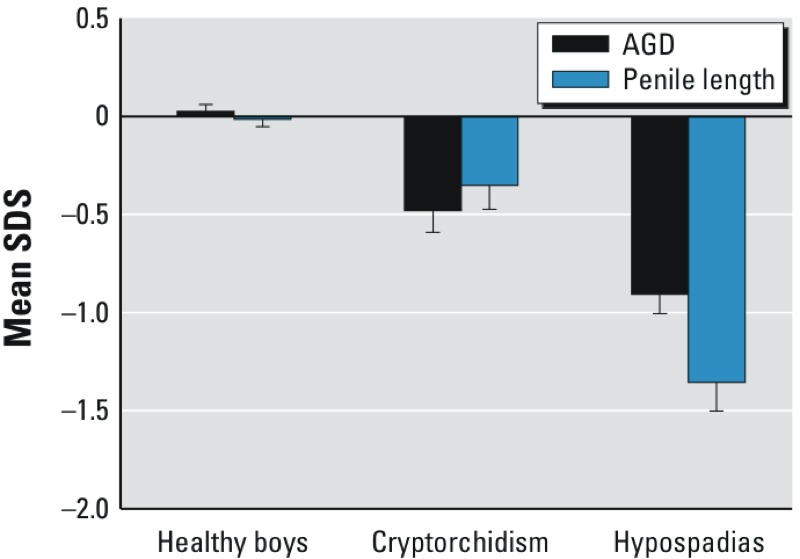
AGD and penile length SDS of healthy boys and those with cryptorchidism or hypospadias. The values of healthy boys were derived from the average of SDS values across multiple visits. Both penile length and AGD were significantly lower in boys with cryptorchidism and hypospadias compared with controls (both *p* < 0.01). Error bars signify SD.

*Hypospadias.* Hypospadias was glanular or subcoronal, penile, or perineal in 51 (62.9%), 22 (27.2%), and 8 (9.9%) boys, respectively. Although five boys with hypospadias had had previous genital surgery, all had penile hypospadias, the surgical repair of which is less likely to affect AGD measurements. The mean age of boys with hypospadias was similar to the age of healthy boys averaged across multiple visits (Table 1). The age distribution suggested two distinct time points for assessment: early infancy (< 6 months) and around 1 year (Figure 1). Boys with hypospadias had lower mean birth weight SDS (*p* = 0.001). They also had reduced current weight SDS compared with the weight SDS of healthy boys averaged across multiple visits, although the difference was not statistically significant (*p* = 0.087). The AGD SDS and penile length SDS of boys with hypospadias were significantly reduced compared with healthy boys (both *p* < 0.0001) (Table 1, Figures 1 and 2). AGD SDS was associated with current weight SDS in the entire cohort of subjects (*r* = 0.18, *p* < 0.0001); however, adjusting for weight SDS did not alter the results (unadjusted data not shown). Increasing severity of hypospadias was associated with a nonsignificant trend for reductions in penile length SDS (glanular or subcoronal: –1.15 ± 1.10; penile: –1.57 ± 1.64; perineal: –2.19 ± 1.01; *p*_trend_ = 0.077) and AGD SDS (glanular or subcoronal: –0.82 ± 0.97; penile: –0.92 ± 0.75; perineal: –1.43 ± 0.87; *p*_trend_ = 0.21).

*Cryptorchidism*. Bilateral undescended testes were present in 13 boys (18.3%) with cryptorchidism. Boys with cryptorchidism (bilateral and unilateral) were assessed over a wider age range than boys with hypospadias, with a peak at 1 year (Figure 1). The mean age of the boys with cryptorchidism was higher than that of healthy boys (*p* = 0.012), the latter derived from the average across multiple visits. Mean values for birth weight, weight at measurement, and body length SDS were similar between the cryptorchid boys and controls. Mean AGD SDS (*p* < 0.0001) and penile length SDS (*p* = 0.002) both were lower in cryptorchid boys than in healthy controls (Table 1, Figures 1 and 2). Mean AGD and penile length SDS were similar in boys with unilateral and bilateral cryptorchidism (data not shown).

*Hypospadias compared with cryptorchidism*. Mean birth weight SDS of boys with hypospadias was lower than that of the boys with cryptorchidism (*p* = 0.051). Boys with hypospadias also had lower mean age (*p* = 0.048) and weight SDS (*p* = 0.037), but mean body length SDS was similar in both groups. Mean AGD SDS (*p* = 0.005) and penile length SDS (*p* < 0.0001) values also were lower for boys with hypospadias than for cryptorchid boys (Table 1, Figures 1 and 2).

*Correlations between AGD and penile length*. In healthy controls, AGD SDS was weakly correlated with penile length SDS (*r* = 0.09, *p* = 0.061). In contrast, AGD SDS and penile length SDS were more strongly correlated among boys with cryptorchidism (*r* = 0.31, *p* = 0.008) and hypospadias (*r* = 0.33, *p* = 0.003).

## Discussion

In this study, mean AGD and penile length parameters were significantly lower in boys with hypospadias or cryptorchidism than in healthy controls from a large birth cohort. Boys with cryptorchidism had intermediate AGD and penile length values, which suggest gradations in the severity of the endocrine disruption that has been hypothesized to underlie these conditions (Dean and Sharpe 2013).

Isolated hypospadias and cryptorchidism are the most common congenital urogenital abnormalities, with an incidence of 0.2–1% and 2–9% respectively (Toppari et al. 2010). Although their etiology remains poorly understood, they share similar risk factors and are postulated to be manifestations at birth of an underlying TDS (Skakkebaek et al. 2001). Large population studies showing higher incidence of hypospadias and cryptorchidism associated with parental exposures to pesticides and other environmental chemicals suggest a role for environmental endocrine disruptors in the pathogenetic mechanisms (Damgaard et al. 2006; Gaspari et al. 2011; Hosie et al. 2000; Toppari et al. 2010; Weidner et al. 1998). Epidemiological links of hypospadias and cryptorchidism with reduced fertility (Skakkebaek et al. 2001), and the observations of reduced insulin-like factor 3 (INSL3) (Bay et al. 2007) and increased gonadotropin levels (Suomi et al. 2006) in cryptorchid boys compared with healthy boys supports an associated testicular dysfunction in these disorders. Furthermore, both conditions are common manifestations of androgen receptor mutations in humans, and can be induced in animals by chemicals that affect testis development or function (Kalfa et al. 2009; Toppari 2008; Toppari et al. 2010).

Based on animal model studies conducted in rats, the reduction in AGD is anti-androgen dose dependent and is associated with outcomes such as reduced penile length, hypospadias, cryptorchidism, and low sperm production (Dean and Sharpe 2013; Gray et al. 1999). A “male programming window” during fetal development (E15.5–E19.5) has also been identified in rats, during which formation of the external genitalia appears to be particularly susceptible to the effects of chemicals acting as endocrine disruptors (Welsh et al. 2008). In contrast to rodents, the genital tubercle in the human is already differentiated as a penis or clitoris at birth. In the present study we used the perineoscrotal junction as the anterior landmark to measure the distance from the anus. It is readily identified and reflects the caudal border of the genital swelling that later differentiates into a scrotum (Salazar-Martinez et al. 2004). The observations of a longer AGD in girls with virilizing congenital adrenal hyperplasia (Callegari et al. 1987) and reports of reduced AGD in adult males with lower testosterone levels (Eisenberg et al. 2012a), semen quality, and fertility rates (Eisenberg et al. 2011, 2012b; Mendiola et al. 2011) support animal experimental data that suggest that this anthropometric measurement represents a long-term biomarker of the effects of fetal testicular function (Dean and Sharpe 2013). Reports of shorter AGD in male infants related to prenatal exposure to environmental chemicals such as phthalates (Suzuki et al. 2012; Swan et al. 2005), bisphenol A (Miao et al. 2011), and dioxins (Vafeiadi et al. 2012) support the utility of this measurement as a biomarker of endocrine disruption in humans.

That the present study has established an association between AGD with congenital genital abnormalities, which have been proposed as important clinical outcomes of TDS, supports the use of the measurement as a biomarker of fetal endocrine disruption. A previous cross-sectional study designed to determine an association of AGD with prenatal phthalate exposure showed that boys with undescended testes had a shorter age-adjusted anogenital index (AGI), the latter calculated as the ratio of AGD to weight (Swan et al. 2005). Another study compared AGD measurements obtained under anesthesia in boys with cryptorchidism (*n* = 32) or hypospadias (*n* = 47) and in 40 controls undergoing surgery for other urological conditions (Hsieh et al. 2008). AGD was reduced in boys with hypospadias. A significant decrease was also observed for boys with cryptorchidism only after adjusting for weight. The subject groups were unmatched for age, but in a subsequent study, age-matched controls of the same racial origin were used, and reduced AGD was found in boys with hypospadias (Hsieh et al. 2012). AGD measurement under anesthesia does have an advantage of increased reliability, but is less suitable for larger epidemiological studies. The advantages of the present study include a larger clinical sample size with healthy controls derived from a well-characterized birth cohort and the application of age-appropriate population-derived normative data. The well-established protocol of measurement was also used in the present study (Salazar-Martinez et al. 2004).

A reduction in both penile length and AGD in boys with hypospadias compared with those with cryptorchidism suggests a more severe and time-specific disruption of genital development in the former group. This is also in keeping with androgen dysfunction occurring during the proposed male programming window in hypospadias, whereas the androgen influence on testis descent occurs during the latter part of gestation (Hughes and Acerini 2008). A positive association between AGD and penile length in cryptorchidism or hypospadias observed in this study suggests that some boys with either of these conditions have more severe reductions in both of these putative androgen sensitive biomarkers. A link between quantitative reductions in AGD and fetal androgen deprivation has been suggested in male reproductive disorders at birth (Dean and Sharpe 2013). Such a potential relationship would need to be tested in studies exploring prenatal exposures and genital disorders at birth.

Previous studies used regression models or estimated AGI to adjust for changes in AGD with age and body size (Hsieh et al. 2008; Swan 2006). However, we observed in an earlier longitudinal study that weight is not related to AGD in older male infants (Thankamony et al. 2009). Furthermore, nonlinear changes in AGD with growth during the first 2 years of life have been reported (Papadopoulou et al. 2013; Thankamony et al. 2009).

These reports highlight the potential advantages of using normative data of AGD in cross-sectional studies. Boys with hypospadias tended to have lower weight compared with controls in the present study, although no differences were observed in previous smaller studies (Hsieh et al. 2011). Nevertheless, the differences in AGD and penile length persisted following adjustment for weight. A potential drawback of the study design was whether a selection bias occurred as we studied referred cases; these may represent more severe forms of genital disorders. There were insufficient data to explore whether the type of cryptorchidism was congenital or acquired, the latter variety accounting for about half of children with cryptorchidism in population studies (Acerini et al. 2009; Wohlfahrt-Veje et al. 2009a).

## Conclusions

In our study population, AGD and penile length were significantly lower in boys with hypospadias or cryptorchidism than in healthy boys. Our observations are based on the study of a relatively large group of subjects, using comparative normative data derived from a contemporary birth cohort study. The findings support the utility of AGD as a biomarker in determining the effects of disruption to androgen function during fetal development (Dean and Sharpe 2013). The supposition that exposure to endocrine disruptors underlies changing trends in common genital birth anomalies can now be tested more reliably in population studies with validated measurements of AGD.

## Supplemental Material

(147 KB) PDF
