# Supplementary material for: Anogenital Distance and Penile Length in Infants with Hypospadias or Cryptorchidism: Comparison with Normative Data
Source: Environ Health Perspect. 2013 Dec 6;122(2):207–11. doi: 10.1289/ehp.1307178 (PMC3915266; doi:10.1289/ehp.1307178)
Supplement: (147 KB) PDF [file ehp.1307178.s001.508.pdf]

Environmental Health Perspectives doi:10.1289/ehp.1307178

Supplemental Material

Anogenital Distance and Penile Length in Infants with Hypospadias or Cryptorchidism: Comparison with Normative Data

Ajay Thankamony, Ngee Lek, Dan Carroll, Martyn Williams, David B. Dunger, Carlo L. Acerini, Ken K. Ong, and Ieuan A. Hughes

Supplemental Material, Table S1. LMS values for anogenital distance and penile length.

	Anogenital distance			Penile length		
**Age (months)**	**L**	**M**	**S**	**L**	**M**	**S**
0	0.546	2.002	0.293	0.278	3.059	0.148
1	0.571	2.210	0.287	0.282	3.157	0.149
2	0.597	2.411	0.280	0.285	3.249	0.150
3	0.623	2.595	0.274	0.288	3.330	0.151
4	0.648	2.754	0.267	0.291	3.398	0.152
5	0.674	2.887	0.261	0.294	3.454	0.152
6	0.700	2.997	0.255	0.297	3.499	0.153
7	0.726	3.085	0.250	0.300	3.537	0.154
8	0.752	3.156	0.245	0.303	3.568	0.154
9	0.777	3.213	0.240	0.306	3.595	0.155
10	0.803	3.257	0.235	0.309	3.619	0.155
11	0.829	3.291	0.231	0.312	3.644	0.155
12	0.855	3.320	0.228	0.315	3.670	0.156
13	0.880	3.344	0.225	0.318	3.699	0.156
14	0.906	3.364	0.222	0.322	3.732	0.156
15	0.931	3.381	0.220	0.325	3.767	0.156
16	0.956	3.395	0.218	0.328	3.803	0.157
17	0.982	3.406	0.217	0.331	3.839	0.156
18	1.007	3.413	0.215	0.334	3.873	0.156
19	1.032	3.418	0.214	0.337	3.904	0.156
20	1.057	3.422	0.213	0.340	3.933	0.155
21	1.081	3.424	0.212	0.343	3.960	0.155
22	1.106	3.426	0.211	0.346	3.984	0.154
23	1.131	3.426	0.211	0.349	4.007	0.153
24	1.156	3.424	0.210	0.352	4.029	0.152

The LMS values can be used to generate standard deviation scores (SDS) for anogenital distance and penile length based on the normative data derived from Cambridge Baby Growth Study. A freely available software, LMSgrowth (Pan and Cole 2012) can be used for this purpose (Cole et al. 2011). The instruction manual included with the software download files provides comprehensive information for deriving SDS using the LMS values.

References

Cole TJ, Williams AF, Wright CM. 2011. Revised birth centiles for weight, length and head circumference in the uk-who growth charts. Annals of human biology 38:7-11.

Pan H, Cole T. 2012. Lmsgrowth, a microsoft excel add-in to access growth references based on the lms method. Version 2.77. Available: www.healthforallchildren.com/?product=lmsgrowth [accessed 22/07/2013 2013].
